# Human milk bank services and Islamic milk kinship: pathways and processes for ensuring respect for religious law and tradition in the provision of donor human milk for small vulnerable newborns

**DOI:** 10.1186/s13006-025-00704-w

**Published:** 2025-04-16

**Authors:** Karleen D. Gribble, Paul Zambrano, Amal Omer-Salim, Mei Chien Chua, Nemat Hajeebhoy, Tuan T. Nguyen, Andini Pramono, Roger Mathisen

**Affiliations:** 1 School of Nursing and Midwifery, Western Sydney University, Parramatta, NSW Australia; 2 Alive and Thrive, FHI 360 Global Nutrition, Manila, Philippines; 3 World Alliance for Breastfeeding Action, Penang, Malaysia; 4 Department of Neonatology, KK Women’s and children’s Hospital, 100 Bukit Timah Road, Singapore; 5 Independent, Mumbai, India; 6 Alive and Thrive, FHI 360 Global Nutrition, Hanoi, Vietnam; 7 College of Health Sciences, VinUniversity, Hanoi, Vietnam; 8 National Centre for Epidemiology and Population Health, Australian National University, Canberra, Australia; 9 Indonesian Breastfeeding Mothers Association, Jakarta, Indonesia

**Keywords:** Donor human milk, Human milk banking, Milk kinship, Small vulnerable newborns

## Abstract

Islam provides strong support for infants to be breastfed, including for wet nursing where mothers are unable to breastfeed. Amongst those infants who may be in need of breastmilk from another woman are small vulnerable newborns. These infants can benefit from donor human milk from a human milk bank (HMB). However, in Islamic contexts, HMBs must be both medically and religiously safe and take account of the religious principle of milk kinship whereby the consumption of breastmilk can create a family relationship between the donor mother and the infant. This paper explores the variety of circumstances under which milk kinship may be created and highlights the two main pathways followed by HMBs to ensure religious safety. It presents the case of the KK HMB in Singapore as an example demonstrating how close collaboration between medical and religious authorities can enable HMBs to provide donor human milk to small vulnerable newborns. Finally, key processes for HMB establishment in the context of Islamic milk kinship are outlined including partnering with key religious leaders, knowing and working with local understandings of milk kinship, ensuring clear communication, proactively addressing community concerns and designing and adapting HMB processes to ensure religious requirements can be maintained.

## Introduction

Islam provides strong support for infants to be breastfed. The Qur’an positions breastfeeding as an expectation for every child for the first two years of their life, with mothers to be supported in their breastfeeding work by the child’s father [1, 2]. There are many verses that support breastfeeding in the Qur’an including, “Mothers shall suckle their children for two years completely, for whoever desires to fulfill the suckling. It is for the father to provide for them and clothe them with kindness” [3]. The Islamic expectation for two-years duration of breastfeeding is aligned with global recommendations from the World Health Organization (WHO) and United Nations Children’s Fund (UNICEF), which state that infants and young children should be breastfed within the first hour of birth, exclusively breastfed for the first six months of life, and continue to be breastfed to two years or beyond [4]. If a mother is unable to breastfeed, Islamic law and tradition support providing a wet nurse [5–8]. This practice also aligns with WHO and UNICEF guidance, which recommends that for those few health situations where infants cannot, or should not, be breastfed by their mother, breast milk from a healthy wet-nurse or a human-milk bank (HMB) are feeding options to be explored [4]. The significance of these breastfeeding practices from a child health and survival perspective is substantial as it is estimated the lives of 600 000 children globally could be saved each year if all infants were breastfed as recommended [9]. Beyond protecting life, the universal application of breastfeeding would guard many more children from serious infections, malnutrition and other adverse health and developmental outcomes [10, 11]. The importance of breastfeeding is thus confirmed from both a religious and medical perspective [7, 12].

## Importance of breastmilk for small vulnerable newborns

Small vulnerable newborns are infants who are born prematurely (before 37 weeks of gestation), are small for gestational age (below the 10th percentile), or have a low birthweight (less than 2500 g) [13]. There are 35 million small vulnerable infants born annually, of which two-thirds are born in southern Asia [13]. These infants are at increased risk of neonatal and post-neonatal death and rehospitalization [14, 15]. They are more vulnerable to serious infections [16, 17] and a bowel condition called necrotising enterocolitis (NEC) [16]. However, through a multitude of means, breastmilk protects against infections [16] and is also highly protective against NEC [18]. Where small vulnerable newborns survive, they are at increased risk of a variety of developmental disorders and lower IQ [19, 20]. However, breastfeeding and breastmilk supports normal child development, including cognitive development [21].

Small vulnerable newborns do not always have access to their mother's own breastmilk. Pregnancy conditions that may contribute to infants being born small or premature such as infection, undernourishment and gestational diabetes may delay the onset of secretion of breastmilk after birth and/or reduce breastmilk production generally [22, 23]. Furthermore, infants who are small or premature are at risk of experiencing difficulties with feeding and may not be able to suckle well at the breast, impeding the establishment of lactation [24]. Finally, mother-infant separation may occur if newborns in need of intensive medical care due to prematurity or illness are transferred to a higher-tier hospital while their mother remains in the maternity facility [25]. Thus, there is often a “breastmilk gap” for small vulnerable newborns [26] and they are at increased risk of not being exclusively breastfed in hospital and following hospital discharge and at are at increased risk of not being breastfed at all times [15].

## Human milk banks protect the lives and health of small vulnerable newborns

In recognition of the importance of breastmilk to small vulnerable newborns, HMBs have been established to protect the health of infants unable to access sufficient maternal breastmilk [27]. HMBs collect, store and dispense expressed breastmilk donated by healthy women with screening of donors, testing of breastmilk, and usually pasteurisation of breastmilk to ensure medical safety [27, 28]. Among premature or low birthweight infants, the use of pasteurised donor human milk (DHM) is associated with a 46% reduction in the incidence of NEC compared with feeding of commercial milk formula (CMF) [18]. Use of pasteurised DHM has also been associated with reduced odds of infants developing sepsis and bronchopulmonary dysplasia and shorter duration of ventilator support and hospital stay compared to feeding preterm CMF [29, 30]. The presence of an HMB and the use of DHM are associated with higher rates of maternal breastfeeding during hospital stays and after hospital discharge, even for infants who are admitted to neonatal intensive care units (NICUs) [29, 31, 32]. The WHO specifically recommends the use of DHM accessed through HMBs for small vulnerable newborns when maternal breastmilk is not available [33]. This recommendation continued to apply during the COVID-19 pandemic [34], although uptake of this recommendation was poor [35], including in Southeast Asia [36].

Yet, up to 40% of newborns, 14 million in total, in NICUs do not have access to sufficient breastmilk from their mother or DHM from an HMB to be exclusively breastfed [37]. Challenges to HMB establishment include the lack of international, regional and national guidelines and technical support, inconsistent regulatory frameworks, as well as the infrastructure requirements for HMBs and the cost for their establishment and operation [28, 37, 38]. In Muslim majority countries the imperative to respect and adjust practice for the Islamic principle of milk kinship has also presented challenges to the establishment and operation of HMBs. This challenge has particular relevance to South and Southeast Asia where 30% and 40% respectively of the population are Muslim [39]. In South and Southeast Asia, there are just 90 HMBs, 85% of these are in just two countries, India and the Philippines and more than 60% of the countries in South and Southeast Asia have no HMBs [40].

## Islamic milk kinship and HMB processes

In Islamic law, breastfeeding is understood not only to provide food and nurturance but also, via breastmilk, to create a kinship relationship between those not otherwise related by blood or marriage. Children who have consumed milk from the same woman may become “milk-brothers and sisters” [41, 42] and they and other relatives made through milk kinship are prohibited from marrying one another [43]. Avoiding consanguineous marriages with relations created through milk kinship is a religious necessity. Therefore, for DHM to be safe from a religious perspective as well as a medical one, HMBs must operate in such a way as to avoid the creation of milk kinship or to enable milk kinship relationships to be considered and consanguineous relationships avoided.

The circumstances under which milk kinship is created vary between Islamic traditions and scholars [44]. For those considering establishing an HMB, it is vital to understand how the operation of an HMB may influence whether and how milk kinship is established in their context and how an HMB may be operated in a religiously safe way. Factors that may determine whether a milk kinship relationship is established include the child's age when they consume the breastmilk, the number of feeds of breastmilk, how the breastmilk is provided, and whether the donor is known or not. Table 1 outlines the variety of circumstances affecting the establishment of milk kinship as reported in the literature.

**Table 1:** The variety of circumstances under which milk kinship may be created, as reported in the literature^1^

Breastmilk is consumed before the child has attained 24 [2, 45] or 30 months [8] of age
The child has any number of breastmilk feeds [45], at least five feeds [8, 45], or at least 15 feeds [2]
The child receives breastmilk over at least 24 h [2]
The child is fed enough breastmilk to reach satiety [2, 46]
The child is fed by breastfeeding directly enabling a bond to be created [2, 45, 47] and not by other means such as bottle, cup or nasogastric tube
The child is fed breastmilk by any means including by direct breastfeeding, bottle, cup or nasogastric tube [45]
The breastmilk reaches the child’s stomach and contributes to their growth [2, 8]
The donor of the breastmilk is known [48]
There is certainty that all of the conditions to create milk kinship have been met including regarding volume of milk, number of milk feeds, and the identify of milk donor [46, 49]

^1^There may be circumstances additional or different from those described here that affect the creation of milk kinship

Considering the different understandings of milk kinship, there are two main pathways that HMBs have followed to ensure medical and religious safety, depending on the context. The first avoids creating milk kinship relationships altogether, while the second enables milk kinship relationships to be documented and consanguineous relationships avoided. While these are the main pathways that have been followed, it important to note that aspects of pathway 1 and pathway 2 may be used in combination with some characteristics mandatory and some optional with “and” or “or” between characteristics (Table 2).

**Table 2:** Characteristics of the two main HMB pathways providing safety in accordance with religious concerns

**Pathway 1: avoiding the creation of milk kinship**	
Pooling of donor human milk from several to many women	Infants are fed only a small amount of milk from several mothers which means that the criteria for milk kinship requiring that the infant be fed to satiety by milk from one woman is not met [48, 50]
Donor human milk is not pooled but is shared between many infants	Infants are fed only a small number of feeds from any one mother meaning that the criteria for milk kinship requiring a certain number of full feeds is not met [46]
Infants receive breastmilk from many women	Infants are fed only a small amount of milk from several to many women which means that the requirement for satiety and/or number of feeds is not met by any single woman [46, 48]
Feeding of breastmilk by means other than direct breastfeeding such as nasogastric tube, bottle or cup	Where the infant does not suckle at the breast, the emotional bonding that occurs during breastfeeding does not occur and bonding criteria for milk kinship is not met [2, 46, 47]
Anonymity of women who have donated to the milk bank	Milk kinship cannot be created if the provider of the breastmilk is unknown [46, 48]
**Pathway 2: recording milk kinship and avoiding consanguineous relationships**	
Breastmilk from multiple donors is not pooled, and infants receive milk from one or few donor mothers who are known	Single or few known donors enables records to be kept of who has donated milk to each infant so that the milk kinship relationships created are few and known and consanguineous relationships can be avoided [49, 51]
System of registration and records kept of the identity of breastmilk donors and breastmilk recipients	Documentation of milk donors and recipiences enables the kinship relationship created by the consumption of breastmilk to be recognised and consanguineous relationships avoided [2, 47, 51]
Introducing milk donor mothers and milk recipient mothers to one another	Where milk donor and milk recipient family know each other, they can ensure that consanguineous relationships are avoided [49, 51, 52] in a similar way to the wet nursing tradition

Pasteurisation of donor human milk in HMBs may provide additional safety assurance for some religious leaders and parents. Pasteurisation of breastmilk disrupts human cells in breastmilk and means that genetic information cannot be transferred from breastmilk to an infant [53].

The context within which breastmilk is obtained and supplied is also extremely important from a religious perspective. HMB practices that undermine women breastfeeding their own infants or the health of donors will not meet religious requirements. Some jurists have expressed concerns that establishment of HMBs would result in women using breastmilk from an HMB rather than breastfeeding their own infants [2]. Others have been troubled about the possibility of impoverished women selling their breastmilk to an HMB while their own infant suffers [2]. HMBs should have strong policies requiring that breastmilk is available only to infants whose mothers are unable to provide them with breastmilk. In addition, the wellbeing of the infants of donor mothers should be prioritised, and donor mothers not paid for their breastmilk. These protective measures must be clearly presented in the dialogue with religious leaders.

Islamic legal scholars also require knowledge of the specific needs and risks faced by small vulnerable newborns. For example, one religious scholar who opposed the setting up of an HMB suggested that cow’s milk or carraway could be fed to newborns instead of DHM seemingly unaware of the danger such feeds would pose [47]. In contrast, religious rulings (fatwa) supporting the establishment of HMBs have recognised that where circumstances are extreme, such as the life of a newborn being at risk, solutions must be found [46]. These rulings note that the prioritisation of the preservation of human life is central to Sharia law [50]. It is therefore important that the extreme situation of small vulnerable newborns, the irreplaceability of breastmilk as a food and medicine and HMBs as a life saving measure are known by religious leaders.

Given the significance of milk kinship, the process of establishing HMBs in Islamic settings requires just as much attention be paid to cultural and religious considerations as medical ones. Formal legal rulings and interpretations of Islamic law in the form of fatwa [54] are the means by which religious safety of HMBs can be endorsed [53]. Obtaining a fatwa is a necessary part of the HMB establishment process. However, recently, a fatwa supporting the operation of an HMB in Pakistan was withdrawn after a loss of confidence in the ability of the HMB to operate according to the procedures outlined in the fatwa [55]. This experience underlines the importance of working closely with religious leaders in the entirety of the planning, operationalising and stabilising and researching and evaluating stages of establishing an HMB [38]. Having the approval of local religious authorities is also needed to dispel any fears of parents of recipient infants who likely share similar concerns. HMBs that are medically and religiously safe have been successfully established where medical authorities have worked closely with religious leaders.

## Case study: KK Human Milk Bank (KKHMB), Singapore

The KKHMB in Singapore provides an example of close collaboration between medical authorities and religious leaders, leading to the establishment of a religiously safe HMB. The KKHMB was opened in 2017 and provides pasteurised DHM to hospitalised small vulnerable newborns whose mothers are unable to provide them with sufficient breastmilk [56]. Since Singapore has 15.6% of the total population following the Muslim religion [57], it was viewed as essential that the HMB operated in a way that aligned with the local understanding of Islamic milk kinship and was safe from a medical and a religious perspective.

At the very beginning of the planning of the HMB, the medical team at the KK Women and Children’s Hospital contacted the Islamic Religious Council of Singapore, Majlis Ugama Islam Singapura (MUIS), for guidance and as a result, a fatwa committee was formed. This committee and the medical team engaged in dialogue about religious and health considerations for HMB services [56]. Through this discussion, the fatwa committee gained an understanding of why there was a desire to set up an HMB. In addition, the medical team gained an understanding of the religious considerations and rules associated with milk kinship in Singapore. This two-way exchange of information made it possible for the team to design an HMB that met the dual outcome of providing medically safe and religiously safe DHM to Muslim infants.

The KKHMB design follows a pathway of avoiding creation of milk kinship and includes:

- Anonymity between donors and recipients
- DHM is fed using a nasogastric tube rather than by mouth
- DHM is not pooled but is shared between infants, each of whom is likely to receive only 2–3 full feeds from any one donor mother
- The amount of milk donated by each donor mother and the number of times the recipient infant receives DHM from the same mother is confidential [46, 56].

The MUIS published a fatwa in support of the HMB, which emphasised not just the processes the KKHMB would take to ensure religious safety but also why DHM was important to small vulnerable newborns, which infants would be eligible to receive DHM and the actions undertaken to ensure the DHM is medically safe. The MUIS made the fatwa publicly available and published frequently asked questions regarding milk kinship. A briefing session was held for Singapore’s Islamic leaders to enable them to support Muslim families regarding the KKHMB [56]. Muslim parents whose infants meet the criteria for DHM are informed about the fatwa and directed to the MUIS website for further guidance if required [56]. Informed consent is obtained before DHM can be dispensed to infants [56]. The KKHMB is a part of the Southeast Asian HMB network, and the HMB staff actively contribute to the network's activities, including hosting learning visits from teams from other countries who want to establish a religiously compliant HMB. During these learning exchanges, it is emphasised that the partnership between the medical team at the KK Women and Children’s Hospital and the MUIS made the KKHMB possible.

## Key processes for HMB establishment in the context of Islamic milk kinship

The following key processes for HMB establishment are proposed based on Islamic principles and the experience of medical teams who have successfully established HMB services in Islamic contexts.

### Partner with key religious leaders from early in the course of considering the establishment of an HMB.

These leaders may be at a local, regional or national level (ideally all). Contact should be made very early, and conversations continue through every stage of planning, implementation, stabilising, and evaluating an HMB service. The focus of the discussion should be to find solutions that enable lifesaving DHM to be available to small vulnerable newborns and ensure safety from a medical perspective while aligning with religious considerations. It is very important to make it clear that circumventing or undermining Islamic law is not being sought but also to ensure the life-threatening situation of small vulnerable newborns and the lifesaving potential of DHM is understood.

### Know and work with local understandings of milk kinship

It is not enough to have a general knowledge of milk kinship or to assume that understanding of milk kinship in one context is generalisable to all. Specifics of the local understandings of how milk kinship is created, and its implications must be known. This vital information is needed to shape the design and implementation of an HMB service and determine whether an approach of avoiding the creation of milk kinship or documenting milk kinship relationships is the best pathway and the specifics within the chosen path. This information can only be obtained through close and careful dialogue with religious leaders and scholars who will also be those who will provide a fatwa for the HMB. Dialogue should be solution-oriented and explore practical options that address salient concerns.

### Ensure clear communication and proactively address community concerns

Clear communication between religious leaders and those planning the HMB is necessary during the planning and implementation. Once a way forward to implement an HMB in a medically and religiously compliant and safe way is identified and a fatwa issued, communication with the community is vital to ensure there is no misunderstanding. It should not come as a surprise to anyone that an HMB is being established. Strategic communication to community members regarding the HMB and its operation should involve both medical and religious leaders. Resources such as frequently asked questions, video explanations and patient hand-out sheets may assist, such as those as developed by the Minnesota Milk Bank for Babies in the USA [58].

### Design and adapt HMB processes to ensure religious requirements can be maintained

Whatever the specific religious requirements are, it is necessary to ensure that HMB processes are implemented to ensure safety. For example, if HMB religious safety relies on the documentation of milk kinship and avoidance of consanguineous relationships, processes must be developed to ensure the reliable recording of breastmilk donors and recipients. If HMB religious safety relies on anonymity between donors and recipients, a process must be in place to prevent donor mothers and recipient families from knowing each other’s identity. An appropriate monitoring system should be in place to ensure sustained compliance with processes outlined in the fatwa for the confidence of both families and religious leaders. While this paper was framed with the situation of South and Southeast Asia at the forefront, the principles described here, apply globally. This includes adapting HMB processes for Muslim infants in Western countries [44, 59–61].

## Conclusion

Establishing HMB services in Islamic contexts requires careful consideration of medical and religious perspectives. The integration of Islamic principles and adaptation of processes to account for milk kinship is crucial to ensure the acceptance and success of HMBs. By partnering with religious leaders, understanding local interpretations of milk kinship, ensuring clear communication, and adapting HMB operations to align with religious safety, it is possible to provide lifesaving DHM to small vulnerable newborns in an Islamic context. In South and Southeast Asia, collaboration between medical authorities and religious authorities is needed to ensure that small vulnerable newborns have access to DHM.

## Data Availability

No datasets were generated or analysed during the current study.

## Abbreviations

DHM: Donor human milk
HMB: Human Milk Bank
MUIS: Majlis Ugama Islam Singapura
